# Outcomes Following Coronary Artery Bypass Graft Surgery in Patients with Mild Preoperative Renal Insufficiency

**DOI:** 10.21470/1678-9741-2017-0148

**Published:** 2018

**Authors:** Weitie Wang, Yuefeng Wang, Rihao Xu, Junwu Chai, Wei Zhou, Honglei Chen, Kai Wang, Xiangrong Kong

**Affiliations:** 1 Department of Cardiovascular Surgery, 1st Central Hospital of Tianjin, Tianjin, China.; 2 Department of Cardiovascular Surgery, 2nd Hospital of Bethune, Jilin University, Changchun, Jilin, China.; 3 Department of Cardiothoracic Surgery, Daqing OilField General Hospital, Daqing, Heilongjiang, China.

**Keywords:** Coronary Artery Bypass, Renal Insufficiency, Glomerular Filtration Rate, Treatment Outcome, Survival Analysis

## Abstract

**Introduction:**

Preoperative renal insufficiency is an independent predictor of mortality after coronary artery bypass graft (CABG) surgery. However, there are few reports aimed to evaluate the impact of mild preoperative renal insufficiency on long-term follow-up outcomes after isolated CABG surgery. This study investigates the effect of mild preoperative renal insufficiency on long-term follow-up outcomes of patients after CABG.

**Methods:**

Five hundred eighty-four patients' data that underwent CABG between 1 January 2009 and 1 December 2016 were retrospectively analyzed. They were divided into two groups: normal group [Estimated glomerular filtration rate (eGFR) ≥ 90 ml/min/1.73 m^2^, n=304] and mild group (eGFR ranges from 60 to 89 ml/min/1.73 m^2^, n=280). Clinical material and long follow-up outcomes were compared inthe two groups.

**Results:**

Two groups had similar baseline and intraoperative data except eGFR. Six (0.01%) patients died in hospital, 15 in normal group and 28 in mild group during the long-term follow-up, which had statistical significance (*P*<0.05). Univariate factor analysis displayed that the two groups had similar in-hospital outcomes. Kaplan-Meier curves showed a better long-term survival in patients with normal preoperative renal function compared to mild preoperative renal insufficiency (*x* ^2^=4.255, *P*=0.039). Cox proportional model presented the hazard ratio of long-term mortality in patients with mild preoperative renal insufficiency compared to normal preoperative renal function was 1.79 (95% CI 1.17-2.88, *P*=0.027).

**Conclusions:**

Patients with mild preoperative renal insufficiency had a higher mortality rate than normal patients in long-term survival, whereas no evidence of worse in-hospital mortality rate was found. Patients with mild preoperative renal insufficiency showed a higher mortality rate than other studies.

**Table:** 

Abbreviations, acronyms & symbols				
AKI	= Acute kidney injury		EuroSCORE	= European System for Cardiac Operative Risk Evaluation
CABG	= Coronary artery bypass graft surgery		GFR	= Glomerular filtration rate
CAD	= Coronary artery disease		HRs	= Hazard ratios
CIs	= Confidence intervals		MACE	= Major Adverse Cardiac Event
CKD	= Chronic kidney disease		MI	= Myocardial infarction
COPD	= Chronic obstructive pulmonary disease		NYHA	= New York Heart Association
CPB	= Cardiopulmonary bypass		OPCAB	= Off-pump coronary artery bypass
CRF	= Chronic renal failure		PCI	= Percutaneous coronary intervention
CT	= Computed tomography		RI	= Renal insufficiency
DM	= Diabetes mellitus		SD	= Standard deviation
ECG	= Electrocardiogram		SVG	= Saphenous vein graft
eGFR	= Estimated glomerular filtration rate			

## INTRODUCTION

Chronic kidney disease (CKD) has been identified as an independent predictor of short- and long-term outcomes after coronary artery bypass graft surgery (CABG)^[1-4]^. Previous studies have paid more attention to moderate and severe preoperative renal insufficiency^[1,5]^. Whereas few studies^[3]^ aimed to evaluate the impact on mild preoperative renal insufficiency (RI) on outcomes after isolated CABG surgery. Thus, this study aims to evaluate the effect of mild preoperative RI on long-term follow-up outcomes of patients underwent CABG.

Nowadays, glomerular filtration rate (GFR) has been recognized as one of the best indicator of renal function^[4]^. It is more objective and accurate and has been changed even serum creatinine is normal. In addition, GFR will not affect by many other factors as serum creatinine. In this study, GFR was used as an index of preoperative renal function to evaluate its influences on in-hospital and long-term outcomes. This study was approved by Clinical Trial Ethics Committee of Tianjin First Central Hospital (Certificate No: E2015011L).

## METHODS

### Patient Selection

Retrospective analysis of 584 patients underwent CABG between 1 January 2009 and 1 December 2016, in the first central hospital of Tianjin, was performed. There were 363 males, with a mean age of 74.17±9.29 years old. Enrollment criteria included the following items: 1. Patient age >65 years old; 2. Normal preoperative renal function and mild preoperative RI (GFR >60 ml/min/1.73 m^2^); 3. Undergoing first isolated CABG surgery. Three hundred and four patients in normal preoperative renal function and 280 patients in mild preoperative RI were selected by these criteria. Baseline and procedural characteristics were shown in Table 1. In comparison to people in normal preoperative renal function group, patients with mild preoperative RI had lower baseline eGFR.

**Table 1:** Baseline and procedural characteristics after matching.

	Normal group (n=304)	Mild group (n=280)	*P* value
Age (years old)	74.11±9.16	73.97±9.55	0.8566
Older age (age >70 years)	169 (55.59%)	162 (57.86%)	0.5810
Older age (age >75 years)	29 (9.53%)	32 (11.43%)	0.4599
Male	181 (59.54%)	182 (65.00%)	0.1741
Obesity (BMI >30 kg/m^2^)	145 (47.70%)	146 (52.14%)	0.2831
Smoking	171 (56.25%)	175 (62.50%)	0.1246
NYHA class III-IV	110 (36.18%)	112 (40.00%)	0.3426
Previous myocardial infarction	99 (32.57%)	97 (34.64%)	0.5954
Previous PCI	78 (25.66%)	76 (27.14%)	0.6841
Hypertension	155 (50.99%)	148 (52.86%)	0.6513
Diabetes mellitus	44 (14.47%)	44 (15.71%)	0.6755
Hyperlipemia	187 (61.51%)	180 (64.29%)	0.4885
COPD	28 (9.21%)	27 (9.64%)	0.8582
Prior cerebrovascular accident	21 (6.91%)	19 (6.79%)	0.9534
Abnormal motion of the segmental cardiac wall	178 (58.55%)	174 (62.14%)	0.3757
Extent of CAD			
Left main stem disease	55 (18.09%)	53 (18.93%)	0.7948
3-vessel	146 (48.02%)	137 (48.93%)	0.8275
2-vessel	103 (33.88%)	90 (32.14%)	0.6554
Baseline eGFR (ml/min/1.73 m^2^)	102.39±10.58	75.14±6.76	<0.0001*
Logistic EuroSCORE	7.7±2.8	7.8±2.3	0.6390

* *P*<0.05

BMI=body mass index; NYHA=New York Heart Association; PCI=percutaneous coronary intervention; COPD=chronic obstructive pulmonary disease; CAD=coronary artery disease; GFR=glomerular filtration rate

Baseline clinical data included age, sex, obesity, smoking, New York Heart Association (NYHA) class, previous myocardial infarction (MI), percutaneous coronary intervention (PCI), diabetes mellitus (DM), hypertension, hyperlipemia, chronic obstructive pulmonary disease (COPD), stroke, prior cerebrovascular accident, abnormal motion of the segmental cardiac wall, eGFR, anatomical severity of coronary artery disease (CAD) and European System for Cardiac Operative Risk Evaluation (EuroSCORE). Operative data included operation time, cross-clamp time, perfusion time, number of distal anastomosis, saphenous vein graft (SVG) and composite graft. Bivariate analyses were performed to examine differences in baseline characteristics between the two groups.

The serum creatinine was measured before surgery and the GFR was calculated by using Cockcroft- Gault formula^[6]^. Normal renal function was defined as eGFR of 90 ml/min/1.73 m^2^ or more and mild RI was defined as eGFR of 60 to 89 ml/min/1.73 m^2^.

The end points studied overall death. Follow-up information was obtained by visit or telephone calls and was agreed by all patients before discharge with informed consent. The mean follow-up time was 72.93±21.25 months.

Clinical outcomes: Surgical mortality was defined as death occurring in hospitalization. Resternotomy for bleeding was defined as reoperation to control bleeding within 36 hours following initial surgery. Postoperative MI was defined by the appearance of new Q waves in two or more contiguous leads on the electrocardiogram (ECG). Atrial/ventricular arrhythmia after off-pump coronary artery bypass (OPCAB) surgery: any episode of atrial/ventricular fibrillation that was registered by the monitoring system on a rhythm strip or the 12-lead ECG. Postoperative respiratory failure (duration of mechanical ventilation more than 72 hours or re-intubation following surgery). Postoperative pneumonia: a positive result in a sputum culture requiring anti-infective treatment, or chest X-ray diagnosis of pneumonia following cardiac surgery. Stroke: new permanent neurological event. Deep sternal wound infection: bone related; any drainage of purulent material from the sternotomy wound and instability of the sternum. Acute kidney injury (AKI) was defined and classified according to the criteria proposed by the Acute Kidney Injury Network. Chronic renal failure (CRF): patients whose GFR declines to 15-20 ml/minute with severe symptoms related to uraemia that can be relieved only by renal replacement therapy. Graft patency was assessed by graft angiography or coronary artery computed tomography (CT) scan, each graft was viewed in at least two orthogonal planes and graded A (excellent), B (fair) or O (occluded) by separate assessment of proximal and distal anastomoses and bypass trunks, graded A and B was designated as patency.

### Statistical Analysis

Continuous data were expressed as a mean ± standard deviation (SD), normally and non-normally distributed continuous variables were compared using a Student t -test and Mann-Whitney U test, respectively. The Kaplan-Meier method was used for determining the overall survival, while the log-rank test was applied for statistical comparison. Univariate Cox regression analysis was used to analysis potential independent predictors. All significant predictors were then entered into a multivariable Cox regression analysis, with entry and retention set at a significance level of *P*<0.05. Hazard ratios (HRs) were reported with 95% confidence intervals (CIs). All statistical analyses were carried out by SPSS 19.0.

## RESULTS

All patients' preoperative characteristics are shown in Table 1. Mean estimated glomerular filtration rate (eGFR) was 102.39±10.58 ml/min/1.73 m^2^ in the normal group and 75.14±6.76 ml/min/1.73 m^2^ in mild group, which has statistical significance (*P*<0.0001). Patients' characteristics except for eGFR have no statistically difference between the two groups.

### Intraoperative Outcomes

Intraoperative data are shown in Table 2. All the operations were operated by the same surgeon. There is no statistical difference between the two groups, including operation time, number of distal anastomosis, perfusion time, cross-clamp time, the SVG use and composite grafting.

**Table 2:** Intraoperative data.

	Normal group (n=304)	Mild group (n=280)	*P* value
Operation time (min)	331±52	333±51	0.6517
No. distal anastomosis	2.44±0.84	2.50±0.80	0.3780
Perfusion time (min)	81.9±42.4	81.8±43.1	0.9775
Cross-clamp time (min)	54.9±24.6	54.8±24.8	0.9610
SVG use	286 (94.08%)	267 (95.36%)	0.4913
LIMA use	198 (65.13%)	192 (68.57%)	0.7807
RITA use	3 (0.99%)	2 (0.72%)	0.7210
Composite grafting	201 (66.12%)	194 (69.29%)	0.4138

SVG=Saphenous vein graft; LIMA=left internal mammary artery; RITA=right internal thoracic artery

### Postoperative Outcomes

All patients were followed up. The mean follow-up time was 72.93±21.25 months. Postoperative outcomes and long-term outcomes are shown in Table 3. Three patients died in normal group due to low output syndrome (Table 4). Three patients died in the mild group, among them, one patient died of malignant arrhythmia and two due to low output syndrome. No significant differences were found between the two groups regarding the number of hospital deaths and postoperative complications.

**Table 3:** Postoperative outcomes.

	Normal group (n=304)	Mild group (n=280)	*P *value
In-hospital			
Surgical mortality	3 (0.99%)	3 (1.07%)	0.9193
Resternotomy for bleeding	1 (0.33%)	1 (0.36%)	0.9535
ICU stay (day)	2.99±1.97	3.32±2.14	0.0528
Hospital stay (day)	9.52±1.50	9.81±2.41	0.0789
Ventricular arrhythmia	1 (0.33%)	1 (0.36%)	0.9535
Low output syndrome	­­	1 (0.36%)	0.2970
Stroke	2 (0.67%)	1 (0.36%)	0.6115
Myocardial infarction	1 (0.33%)	1 (0.36%)	0.9535
Atrial fibrillation	216 (71.05%)	208 (74.29%)	0.3815
IABP support	7 (2.30%)	10 (3.57%)	0.3622
AKI requiring dialysis	2 (0.66%)	3 (1.07%)	0.5879
Respiratory failure	__	1 (0.36%)	0.2970
Pneumonia	10 (3.29%)	9 (3.21%)	0.9592
DSWI	6 (1.97%)	5 (1.79%)	0.8674
CRF requiring dialysis	__	1 (0.36%)	0.2970
Long-term			
Mortality	15 (4.93%)	28 (10.00%)	0.0192*
Graft patency	259 (85.20%)	199 (71.07%)	<0.0001*
Additional PCI after CABG	15 (4.93%)	26 (9.29%)	0.0398*

* *P*<0.05

ICU=intensive care unit; IABP=intra-aortic balloon pump; AKI=acute kidney injury; DSWI=deep sternal wound infection; CRF=chronic renal failure; PCI=percutaneous coronary intervention; CABG=coronary artery bypass graft

**Table 4:** Causes of death.

	Normal group	Mild group
In-hospital		
No. Patients	3	3
Low cardiac output	3	2
Malignant arrhythmia	__	1
Long-term		
No. Patients	15	28
MACE		
Heart failure	1	4
Myocardial infarction	2	12
Ventricular fibrillation	1	4
Sudden death	1	3
Cancer	6	2
Stroke	4	1
Renal failure need hemodialysis	__	2

MACE=major adverse cardiac events

Forty three patients died during long-term follow-up, with a long-term survival rate of 92.64%. Patients with mild preoperative RI had a higher long-term mortality in comparison to normal preoperative renal function (10% *vs.* 4.93%, *P*<0.05). Cardiac deaths (major adverse cardiac event - MACE) occurred in five patients in the normal group and 23 patients in mild group which has a significant difference (*P*<0.05). Four deaths in the mild group were caused by heart failure, and 12 were caused by MI. No significant difference in surgical mortality was found between the two groups (0.99% *vs.* 1.07%, *P*>0.05).

**Fig. 1:**
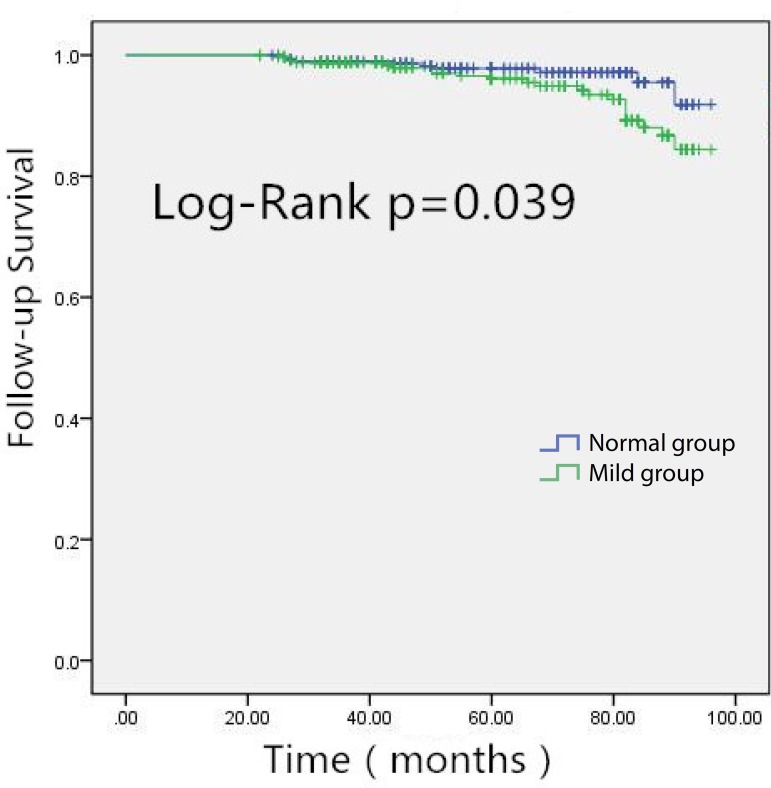
Kaplan-Meier curves showing better long-term survival in patients with normal pre-operative renal function in comparison to mild preoperative renal insufficiency (χ^2^=4.255, P=0.039).

During the longer follow-up, Kaplan-Meier curves (Figure 1) displayed a better long-term survival in patients with normal preoperative renal function than mild preoperative RI (χ^2^=4.255, *P*=0.039). Cox regression revealed that GFR was a significant variable related to the long-term survival. After the Cox proportional model was used, the HR of long-term mortality in patients with mild preoperative RI was 1.78 (95%CI 1.18-2.87, *P*=0.026; Table 5).

**Table 5:** Predictors of long-term mortality.

Variable	HR	95% CI	*P* value
Grouping (mild group vs. normal group)	1.78	1.18–2.87	0.026
Diabetes mellitus	1.54	1.15–2.64	0.005
Prior cerebrovascular accident	1.36	1.08–2.20	0.02
Gender (female *vs.* male)	1.28	1.09–1.99	0.024
Impaired left ventricular function	1.21	1.06–1.68	0.019
Age (per year)	1.07	1.01–1.54	<0.0001

## DISCUSSION

Previous studies have considered RI as an individually risk factor associated with long-term mortality after CABG. Zhang' et al.^[1]^ study showed that RI patients with a normal serum creatinine level adverse clinical outcomes after revascularization especially Chinese older females. Holzmann et al.^[5]^ found that severe renal dysfunction will lead to an increased risk of cardiac events of women in Sweden. However, if mild RI will influence the survival after CABG, up to now, there is still little data report about this risk factor on patients after CABG. In our single-centre cohort study of 584 patients that underwent isolated CABG, there were significantly higher 5-year mortality rates of patients that had mild preoperative RI compared with patients without preoperative RI.

Serum creatinine has been used as an indicator for evaluating of renal function for a long time^[7]^. Nowadays, GFR calculated from the Cockcroft and Gault study equation is considered as the best overall measure to categorize the preoperative renal dysfunction. At the same time, the EuroSCORE including patients' age and creatinine as variables is widely used for risk evaluation in cardiac surgery^[8]^. Hence, a multivariate model using the EuroSCORE could reduce the ability to detect the effect of eGFR and might underestimate the long-term risk.

Our retrospective study shows patients with mild preoperative RI reduced the long-term survival compared with normal renal function after CABG surgery. Univariate factor analysis reveals that these patients compared with normal preoperative renal function had lower long-term survival (95.9% *vs.* 91.6%, *P*<0.05), and Kaplan-Meier curves shows a lower postoperative long-term survival in older patients compared with normal preoperative RI (Log-Rank χ^2^= 4.255, *P*=0.039). Cox regression reveals that preoperative renal function (mild insufficiency *vs.* normal) is related to the long-term survival which has a statistical significant and the HR of long-term mortality in patients with mild preoperative RI was 1.72 (95% CI 1.06-2.83, *P*=0.032). In our study, all patients' age is greater than 65 years old. However, the age, as a HR for long-term mortality, shows no difference with other studies^[7,9-12]^.

Howell et al.^[7]^ performed a large-scale study containing 7621 patients undergoing heart operation and analysed in-hospital mortality and late survival outcome. They concluded that mild preoperative renal dysfunction is an important independent predictor of in-hospital and late mortality in adult patients undergoing cardiac surgery. Jyrala et al.^[9]^ conducted a study of about 885 patients with or without mild preoperative renal dysfunction undergoing on-pump cardiac surgery, with respect to short- and long-term outcomes. They found a mild increase in serum creatinine was a marker for patients with increased cardiac risk factors and the risk of poor outcomes. Ji et al.^[10]^ showed that mild preoperative RI has impact on in-hospital and long-term outcomes after off-pump CABG.

Garg et al.^[12]^ report had proved that off-pump or on-pump CABG surgery had no significant difference in the loss of kidney function within 1 year. Why mild preoperative RI decreased long-term mortality after CABG surgery is still being investigated. Recently, Günday et al.^[12]^ reported that patients with mild preoperative RI have a significantly lower mean coronary flow reserve after CABG surgery compared with normal preoperative renal function (2.09±0.08 *vs.* 2.37±0.06, *P*<0.05). Then they concluded that mild RI can produce adverse effects due to deterioration of the microvascular bed.

In the present study, the long-term mortality is higher than other series^[7,9,10,12]^. Although our operation was assisted by cardiopulmonary bypass (CPB), the ROOBY trial^[13]^ that compared off-pump *versus* on-pump CABG, did not show a benefit of OPCAB for postoperative renal function. In addition, several studies showed that the OPCABG will not influence the long-time survival comparing with CABG in patients such as thin vessels, intramyocardial way, or severe atherosclerosis^[14-16]^. Thus, the most probable reason is that the mean age of patients in our study is higher than other studies and patients' multiple organs function is lower than the patients in other studies during CABG.

### Limitation

This study has several limitations. Firstly, this study was a retrospective observational study with a single central which may influence the generalizability. A final determination would need a prospective, multi-centre study with larger sample size. Secondly, the Cockcroft-Gault formula in this report is not the gold standard for determining GFR although it has been seemed as an acceptable estimate of GFR and preoperative eGFR may fluctuate particularly in patients with unstable hemodynamics. Thirdly, although reports have proved that CPB will not influence kidney function in a short time, more negative impacts on the postoperative renal function for a long time is not confirmed and no data were available on medications taken after discharge because of postoperative treatment from different hospitals. Finally, the mean years old in this study is older than other reports while patients older than 75 years old in this study are still a small part of all patients.

## CONCLUSION

Older patients with mild preoperative RI had a higher mortality rate than normal patients in long-term survival, whereas no evidence of worse in-hospital mortality rate was found. Older patients with mild preoperative RI showed a higher mortality rate than other studies.

**Table:** 

Authors' roles & responsibilities	
WW	Conceived the study, and participated in its design and coordination and helped to draft the manuscript; final approval of the version to be published
YW	Conceived the study, and participated in its design and coordination and helped to draft the manuscript; final approval of the version to be published
RX	Conceived the study, and participated in its design and coordination and helped to draft the manuscript; final approval of the version to be published
JC	Carried out the data collection and statistical analysis; final approval of the version to be published
WZ	Carried out the data collection and statistical analysis; final approval of the version to be published
HC	Carried out the data collection and statistical analysis; final approval of the version to be published
KW	Participated in the design of the study and drafted the manuscript; final approval of the version to be published
XK	Participated in the design of the study and drafted the manuscript; final approval of the version to be published; final approval of the version to be published
